# The Interaction between Zein and Lecithin in Ethanol-Water Solution and Characterization of Zein–Lecithin Composite Colloidal Nanoparticles

**DOI:** 10.1371/journal.pone.0167172

**Published:** 2016-11-28

**Authors:** Lei Dai, Cuixia Sun, Di Wang, Yanxiang Gao

**Affiliations:** Beijing Advanced Innovation Center for Food Nutrition and Human Health, Beijing Laboratory for Food Quality and Safety, Beijing Key Laboratory of Functional Food from Plant Resources, College of Food Science & Nutritional Engineering, China Agricultural University, Beijing, P. R. China; Universidade Estadual Paulista Julio de Mesquita Filho, BRAZIL

## Abstract

Lecithin, a naturally small molecular surfactant, which is widely used in the food industry, can delay aging, enhance memory, prevent and treat diabetes. The interaction between zein and soy lecithin with different mass ratios (20:1, 10:1, 5:1, 3:1, 2:1, 1:1 and 1:2) in ethanol-water solution and characterisation of zein and lecithin composite colloidal nanoparticles prepared by antisolvent co-precipitation method were investigated. The mean size of zein-lecithin composite colloidal nanoparticles was firstly increased with the rise of lecithin concentration and then siginificantly decreased. The nanoparticles at the zein to lecithin mass ratio of 5:1 had the largest particle size (263 nm), indicating that zein and lecithin formed composite colloidal nanoparticles, which might aggregate due to the enhanced interaction at a higher proportion of lecithin. Continuing to increase lecithin concentration, the zein-lecithin nanoparticles possibly formed a reverse micelle-like or a vesicle-like structure with zein in the core, which prevented the formation of nanoparticle aggregates and decreased the size of composite nanoparticles. The presence of lecithin significantly reduced the ζ-potential of zein-lecithin composite colloidal nanoparticles. The interaction between zein and lecithin enhanced the intensity of the fluorescence emission of zein in ethanol-water solution. The secondary structure of zein was also changed by the addition of lecithin. Differential scanning calorimetry thermograms revealed that the thermal stability of zein-lecithin nanoparticles was enhanced with the rise of lecithin level. The composite nanoparticles were relatively stable to elevated ionic strengths. Possible interaction mechanism between zein and lecithin was proposed. These findings would help further understand the theory of the interaction between the alcohol soluble protein and the natural small molecular surfactant. The composite colloidal nanoparticles formed in this study can broaden the application of zein and be suitable for incorporating water-insoluble bioactive components in functional food and beverage products.

## Introduction

Food macromolecules and small molecular compounds are usually used to fabricate food-grade colloidal structures such as colloidal nanoparticles, complexes, and microcapsules, which show good potential for applications in food and beverage industries [1].

Zein, the major storage protein of corn, is generally regarded as safe (GRAS) food ingredient by the US Food and Drug Administration and it is a hydrophobic protein which can be solubilized in high concentration ethanol aqueous solutions (60–90%) but insoluble in water [2]. Because of the difference in soluble characteristics, zein can be easily converted into spherical colloidal nanoparticles by the antisolvent precipitation method, which makes it to be an ideal delivery system for drugs and micronutrients in food, pharmaceutical and biotechnological products [3–4]. Based on this property, zein has been widely used to fabricate colloidal delivery systems for encapsulation of nutraceuticals and functional ingredients such as lutein [5], curcumin [5–7] and quercetin [8].

However, due to the high surface hydrophobicity and low net charge near the isoelectric point (pI ≈ 6.2) of zein nanoparticles, the physical stability of zein nanoparticles was not ideal. To stabilize hydrophobic zein nanoparticles, several biopolymers such as chitosan, sodium alginate and sodium casein have been utilized as stabilizers to prevent the aggregation of zein nanoparticles by reducing the surface hydrophobicity, or increasing electrostatic and steric repulsion [2, 9]. Furthermore, some small molecule surfactants have also been found to form stable complexes with zein by reducing hydrophobic attraction and increasing steric repulsion. Tween80 was used as a stabilizer to form a core–shell structure with zein, which consisted of a zein core with a diameter around 78 nm and a surfactant shell with a thickness around 4 nm [10]. The zein and sodium stearate complexes, which were generated mainly due to nonspecific hydrophobic interaction, resulting in the partial unfolding of zein particles and the exposure of hydrophobic microdomains, improved the diffusive mobility of water-insoluble zein particles and endowed the particles with equilibrium interfacial wetting properties [11]. Moreover, a combination of lecithin and Pluronic F68 was reported to stabilize the zein nanoparticles [12].

Lecithin, a kind of natural small molecular surfactants, is widely applied in food, pharmaceutical, and cosmetic industries. Soy lecithin prepared from soybeans can delay aging, regulate blood lipid, lower cholesterol, enhance memory, prevent and treat diabetes [13]. As lecithin consists of a glycerol backbone esterified with two fatty acids and a phosphate group, it has an excellent emulsifying property and is widely applied as an emulsifier in food-grade emulsions. Interactions between proteins and phospholipids may cause the changes in the surface activity, modification on the protein structure and net charge, and incorporation of protein into surfactant micelles and vesicles [14]. The presence of lecithin could enhance the initial characteristics of protein emulsions (high density of small and low flocculated particles) and diminish the creaming rate [15]. Kongsawat and Klinkesorn [16] studied the interaction between lecithin and whey protein concentrate (WPC) in aqueous solution, the result indicated that WPC could interact with lecithin and form the insoluble complex through electrostatic attraction at pH 3.0. Li et al. [17] also investigated the binding mechanism of lecithin to soybean 11S and 7S globulins and the result showed that fluorescence of 11S and 7S was quenched by lecithin due to the formation of a lecithin-globulins complex. The gelatin and lecithin could also form a complex and have the synergistic surface activity, which effectively prevented the change of thymol nanodispersions particle size due to coalescence and Ostwald ripening [18].

To the best of our knowledge, there is no information on the interaction between zein and lecithin in ethanol-water solution. The objective of this work was to investigate the influence of natural small molecular surfactant (soy lecithin) on the physical, structural, thermal and morphological characteristics of zein and lecithin composite colloidal nanoparticles. The interaction between zein and lecithin was characterized by particle size, zeta potential, fluorescence spectroscopy, circular dichroism (CD), fourier transform infrared spectroscopy (FTIR), differential scanning calorimetry (DSC) and atomic force microscopy (AFM). The zein-lecithin complex might have a great potential to incorporate bioactive compounds, which would expand the application of water-insoluble proteins in food industry.

## Materials and Methods

### 2.1. Materials

Zein with a protein content of 91.3% (w/w) was purchased from Sigma-Aldrich, Inc. (St. Louis, MO, USA). Soy lecithin (S-100, 94% phosphatidylcholine) was from Lipoid (Germany). Absolute ethanol (99.9%) was acquired from Eshowbokoo Biological Technology Co., Ltd. (Beijing, China).

### 2.2. Preparation of Zein and Lecithin Mixed Solutions

Briefly, 1.0 g zein was dissolved in 100 mL 70% (v/v) ethanol–water solution with magnetic stirring at 600 rpm for 2 h. Then different quantities of soy lecithin were added to the zein solution at zein to lecithin mass ratios of 20:1, 10:1, 5:1, 3:1, 2:1 1:1 and 1:2, respectively, under continuous stirring (600 rpm) for 3 h. The mixed solutions were allowed to stand for 2 h at room temperature to form the nanoparticles, and fluorescence spectroscopy and far-UV CD were analysed.

### 2.3. Preparation of Zein and Lecithin Composite Colloidal Nanoparticles

The formation of composite colloidal particles was induced by the antisolvent co-precipitation method described in previous literature with some modifications [19]. Briefly, 20 mL zein or zein-lecithin solution prepared as described above was slowly injected into 60 mL deionized water, using a syringe with a constant stirring at 600 rpm for 30 min. The zein and zein-lecithin composite nanoparticle dispersions were acquired. Finally, the dispersions were stored in the refrigerator at 4°C for further analysis, and part of the dispersions were freeze-dried for 48 h with Alpha 1–2 D Plus freeze-drying apparatus (Marin Christ, Germany) to acquire dry particles for solid state characterization analysis.

### 2.4. Particle Size, Polydispersity Index (PDI), Zeta (ζ)-Potential and Turbidity Measurements

The particle size, PDI and ζ-potential were determined by dynamic light scattering (DLS) measured using a Zetasizer Nano-ZS90 (Malvern Instruments Ltd., Worcestershire, UK). The colloidal dispersions were diluted to a suitable volume with MilliQ water at room temperature before the measurement to avoid multiple particle effects. The particle size data were reported as cumulative mean diameter (size, nm). All measurements were carried out at 25°C, and the results were reported as averages of three readings.

Nephelometry experiments were performed in a HACH 2100N laboratory turbidimeter (Loveland, USA), and the turbidity of zein and lecithin composite colloidal dispersions was evaluated as described by Sun et al. [20]. The optical apparatus was equipped with a tungsten-filament lamp with three detectors: a 90° scattered-light detector, a forward-scatter light detector, and a transmitted light detector. The calibration was performed using a Gelex Secondary Turbidity Standard Kit (HACH, Loveland, USA), which consists of stable suspensions of a metal oxide in a gel. All experiments were performed in triplicate.

### 2.5. Fluorescence Spectroscopy Analysis

Fluorescence measurements were carried out using a fluorescence spectrophotometer (F-7000, Hitachi, Japan) according to the description of Joye et al. [21] with some modification. Scanning parameters were optimized with the slit width of 20 nm for excitation and 10 nm for emission. The excitation wavelength was set at 280 nm to selectively excite the tryptophan residues. The zein and lecithin dispersions were diluted to a constant zein concentration (0.2 mg/mL) with 70% ethanol–water solution. The emission spectra collected at 290 and 450 nm with a scanning speed of 100 nm/min. Fluorescence emission spectrum of zein was recorded with the excitation wavelength at 280 nm. Both excitation and emission slit widths were set at 10 nm. Each individual emission spectrum was the average of three runs. All data were collected at room temperature.

### 2.6. CD Measurement

Far-UV CD spectra were recorded between 190 and 260 nm using a CD spectropolarimeter (Pistar π-180, Applied Photophysics Ltd., U.K.). Path length was 0.1 cm and a constant nitrogen flush was applied during data acquisition. The collected data were analyzed using Dichroweb: the online Circular Dichroism Web site http://dichroweb.cryst.bbk.ac.uk [22]. The fractions of α-helix, β-sheet, and unordered coil were estimated by K2D-SET4. Each spectrum was the average of three consecutive measurements.

### 2.7. FTIR

The chemical structures of zein colloidal particles in the absence and presence of lecithin were monitored by a Spectrum 100 Fourier transform spectrophotometer (Perkin-Elmer, UK). Briefly, 2.0 mg sample was mixed with 198 mg pure potassium bromide (KBr) powders. Then the mixture was ground into fine powder, pressed into pellet and measured by FTIR. The spectra were acquired at wave numbers of 400–4000 cm^-1^ in 64 scans with a resolution of 4 cm^-1^. Pure KBr powder was used as a baseline. The data were analyzed using the software OMNIC 8.2 (Thermo Fisher Scientific Inc., Waltham, MA, USA).

### 2.8. DSC

The thermal characteristics of zein and zein-lecithin composite nanoparticles were studied using differential scanning calorimetry (DSC-60, Shimadzu, Tokyo, Japan). Approximate 3 mg of each freeze-dried sample was weighted in an aluminum pan and hermetically sealed. The lids were pinned through with a syringe needle to exclude the interference of moisture. An empty sealed aluminum pan was applied as baseline. Samples were heated from 30 to 150°C at 10°C/min with a constant purging of dry nitrogen at a rate of 20 mL/min.

### 2.9. AFM

The morphology of colloidal nanoparticles was observed by AFM (DI Nanoscope TV, Veeco Company, Plainview, NY) equipped with an E-scanner. Tapping mode with nominal spring constant of 20–100 N m^-1^ and nominal resonance frequencies of 10–200 kHz were employed. The diluted samples were immediately spread evenly onto freshly cleaved mica sheets mounted on sample disks with great care and air-dried for more than 3 h before AFM scans were taken.

### 2.10. Stability of Zein and Lecithin Composite Nanoparticles

#### 2.10.1 Effect of salt

The effect of salt on the stability of zein and lecithin composite nanoparticles was evaluated according to the method of Chen et al. [23] with some modifications. The colloidal dispersions were diluted with the same volume of sodium chloride solution to form a range of different salt concentrations (0 to 300 mM NaCl). The samples were stored at 4°C overnight.

#### 2.10.2 Effect of temperature [10]

The zein and zein-lecithin composite nanoparticles dispersions were incubated in water bath for 30 min (50–90°C) and cooled down to ambient temperature. The particle size of the samples was measured.

#### 2.10.3 Effect of pH

The effect of pH on the stability of colloidal nanoparticle dispersions was according to the method of Hu and McClements [2] with some modification. Approximately 10 mL colloidal dispersions were mixed with equal volumes of 10 mM citric acid–phosphate buffers with different pH values (4.0, 5.0, 6.0, 7.0, 8.0). And the samples were adjusted to the desired pH using 1mol/L NaOH or HCl. The colloidal dispersions were mixed with equal volumes of 10 mM citric acid solution and adjusted onto pH 2.0 and 3.0 by 1 mol/L HCl. The particle size andζ-potential of samples were determined.

### 2.11. Statistical Analysis

All the data obtained were average values of triplicate determinations and subjected to statistical analysis of variance using SPSS 18.0 for Windows (SPSS Inc., Chicago, USA). Statistical differences were determined by one-way analysis of variance (ANOVA) with Duncan’s post hoc test and differences were considered to be significant with p < 0.05.

## Results and Discussion

### 3.1. Particle Size, PDI, ζ-Potential and Turbidity

The antisolvent co-precipitation approach was used to fabricate zein and lecithin composite colloidal particles and both of zein and lecithin were dissolved in 70% (v/v) aqueous ethanol solution. It was different from the traditional antisolvent method, by which zein in ethanol-water solution was directly injected to lecithin aqueous solution.

**Fig 1:**
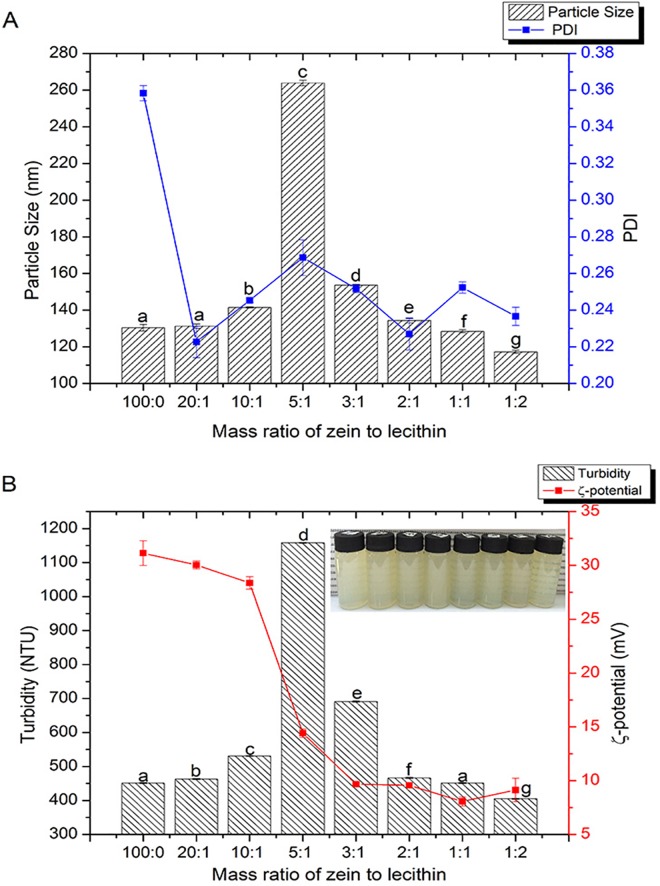
Effect of zein and lecithin mass ratios on particle size (A), PDI (A), turbidity (B) and ζ-potential (B) of the composite colloidal nanoparticle dispersions. Values with different letters are significantly different (p < 0.05).

The influence of lecithin concentration on the particle size and PDI of the zein nanoparticles was shown in Fig 1A. The mean size of zein nanoparticles was 130 nm (Fig 1A). When the zein and lecithin mass ratio was ≤ 5:1, the size of zein-lecithin composite colloidal nanoparticles was markedly (*p<0*.*05*) increased from 130 to 263 nm as the lecithin concentration increased. However, continuing to increase the lecithin concentration (zein and lecithin mass ratio at 3:1, 2:1, 1:1, 1:2) led to a significant (*p<0*.*05*) decrease in the particle size of composite nanoparticles. The zein and lecithin composite colloidal nanoparticles at the mass ratio of 5:1 had the largest size (263 nm). This phenomenon might be because that at a lower lecithin concentration (zein and lecithin mass ratio ≤ 5:1), the zein and lecithin could form composite colloidal nanoparticles, nevertheless they might aggregate due to the increased interactions at a higher proportion of lecithin. When the zein and lecithin mass ratio was more than 5:1, the zein and lecithin composite colloidal nanoparticles might form a reverse micelle-like or a vesicle-like structure with zein in the core, which decreased the particle size of zein-lecithin colloidal nanoparticles. Compared with zein nanoparticles, the PDI values of zein-lecithin colloidal nanoparticles were decreased after adding lecithin. The PDI values were 0.223, 0.245, 0.269, 0.252, 0.227, 0.252 and 0.237 for samples of zein to lecithin ratios at 20:1, 10:1, 5:1, 3:1, 2:1, 1:1, 1:2, respectively, suggesting acceptable homogeneity. Zhou et al. [24] reported that the lecithin-insulin complex with the mass ratio of 7.5:1, preparing by antisolvent co-precitation approach, formed a reverse micelle-like structure with the insulin in core. Gao et al. [11] also suggested that the hydrodynamic size, polydispersity index (PDI), and ζ-potential of the zein colloidal particles decreased sharply with increasing sodium stearate concentration up to 5 mM and then remained constant until 20 mM, which indicated the existence of a critical complexation concentration of sodium stearate (about 5 mM).

The turbidity of the colloidal nanoparticle dispersions was shown in Fig 1B. Nephelometry was used to obtain some information about the physical–chemical driving forces involved in the formation of zein and lecithin composite nanoparticles. Turbidity was affected by many factors such as the particle size, ζ-potential, refractive index, color of the sample and particle interactions including micro- and nano-aggregation [25]. As shown in Fig 1B, the turbidity of the zein colloidal dispersion was 451 NTU, and the presence of lecithin significantly (*p < 0*.*05*) increased the turbidity of zein-lecithin composite colloidal dispersion to 463, 531 and 1158 NTU at zein and lecithin mass ratios of 20:1, 10:1, and 5:1, respectively. Continuing to increase the level of lecithin, the turbidity of zein and lecithin composite colloidal dispersions was greatly (*p<0*.*05*) decreased. The change of turbidity could be also observed according to the illustration in Fig 1B. And the alteration trend of turbidity was consistent with that of particle size. The increased turbidity might be attributed to the micro- and nano-aggregation of zein and lecithin composite colloidal nanoparticles due to the increased interaction at a higher proportion of lecithin. The decreased turbidity might be ascribed to the fact that excessive lecithin molecules were bound to the surface of zein and lecithin composite colloidal nanoparticles, forming vesicle-like structure and inhibiting the aggregation of nanoparticles.

The presence of naturally small molecular surfactant (lecithin) exhibited a significant effect on the electrical characteristics of the zein and lecithin composite colloidal nanoparticles (Fig 1B). In the absence of lecithin, the ζ-potential of zein nanoparticles was +31 mV due to ionisation of amino acids. Patel et al. [26] also reported that zein colloidal particles prepared by precipitation process in water had positively charged surface. After the addition of lecithin, the ζ-potential was significantly (*p<0*.*05*) decreased from +31 mV to +30 mV, +28 mV, +14 mV and +9 mV, for zein and lecithin composite colloidal particles at the ratios of 20:1, 10:1, 5:1, and 3:1, respectively. Further increasing the lecithin concentration (zein to lecithin ratios of 2:1, 1:1, 1:2) showed rarely impact on the ζ-potential of the composite colloidal nanoparticles. This decrease in positive charge might be ascribed to the fact that lecithin could interact with the cationic zein molecules. Hu and McClements [10] also reported that the positive charge of the zein nanoparticles was decreased in the presence of Tween 80. The decreased charge was attributed to Tween 80, which was slightly negative at pH 4, adsorbed to the cationic zein nanoparticle. Moreover, McClements [27] also interpreted that the adsorption of a layer of non-ionic surfactants might decrease the measured electrical potential of nanoparticles by increasing the plane of shear, which was the distance from the particle surfaces where any counter-ions remained strongly attached when the particles moved through an electrical field. The decreased charge on the surface of zein-lecithin composite nanoparticles was another reason for causing the increased particle size.

### 3.2. Fluorescence Spectroscopy Analysis

**Fig 2:**
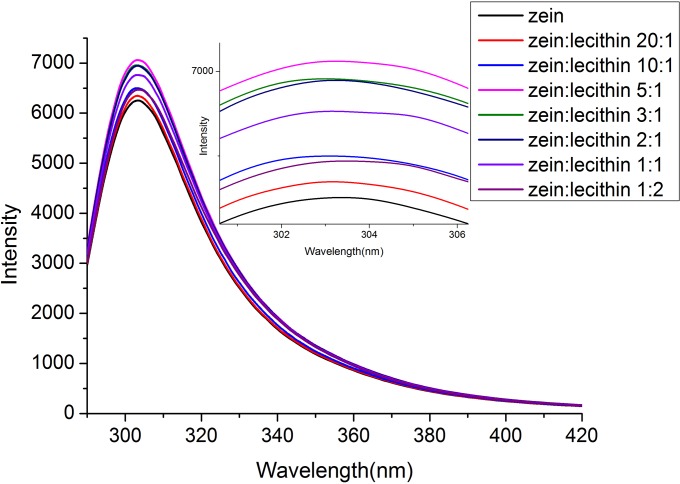
Fluorescence spactra of zein and zein-lecithin complex in 70% (v/v) ethanol-water solution.

The fluorescence emission spectra of zein and zein-lecithin complex in 70% ethanol-water solution were presented in Fig 2. Fluorescences of proteins are originated from tyrosine (Tyr), tryptophan (Trp), and phenylalanine (Phe) residues. The native zein exhibited a strong fluorescence emission peak at 304 nm after being excited at 280 nm, which was attributable to its amino acid composition. Zein contains a high level of tyrosine residues (about 5.0% w/w), which has a typical emission maximum around 304 nm [21]. When the zein to lecithin mass ratio was ≤ 5:1, the intensity of the fluorescence emission of zein at around 304 nm was gradually increased with the rise of lecithin level. This result might be ascribed to the interaction between zein and lecithin, which resulted in the exposure of more chromophores (tyrosyl groups) on the molecular layer. Li et al. [17] also revealed that the addition of lecithin to globulin solution modified the protein structure. The fluorescence intensity of zein and lecithin complex began to decrease as the lecithin concentration further increased. This result was consist with the report of Maikokera and Kwaambwam [28], who found that low concentration of sodium dodecyl sulfate (SDS) resulted in a large increase in the fluorescence intensity of the protein extracted from *Moringa oleifera* seeds. However, the intensity began to decrease with SDS increasing, which was attributed to the tryptophan environment in the coagulant protein changed as SDS was added and this might be interpreted as a change in the tertiary structure of the protein. On the contrary, the fluorescence intensity of globulin was decreased after lecithin addition, indicating that lecithin quenched the intrinsic fluorescence of globulins by interaction [16].

### 3.3. CD Measurement

**Fig 3:**
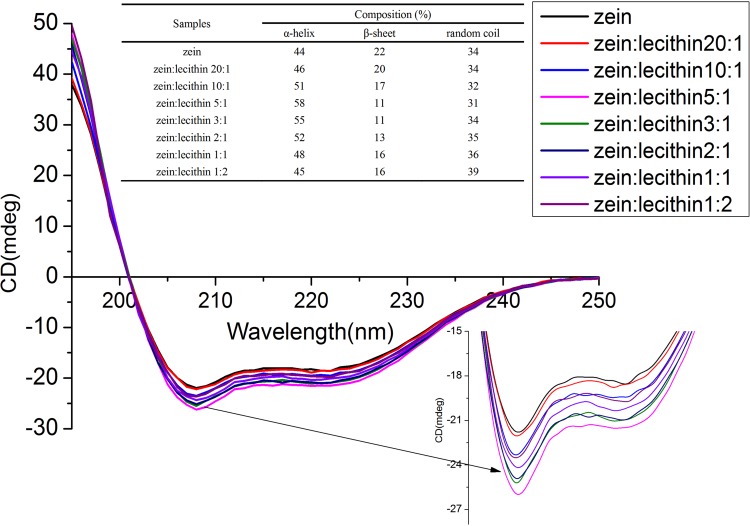
CD spectra of zein and zein-lecithin complex in 70% (v/v) ethanol-water solution.

The secondary structures of zein and zein-lecithin complex in 70% ethanol-water solution were measured using far-UV CD spectroscopy. As shown in Fig 3, the spectra of zein showed two negative peaks at 208 and 221 nm, which corresponded to α-helical structure. This kind of α-helical spectrum was very similar to that published earlier [29]. CD ellipticity at 221 nm was widely used to estimate α-helix content in the protein structure [30]. In the presence of lecithin, the spectra of zein was changed in ellipticity at 221 nm, which indicated that the interaction between lecithin and zein significantly modulated the secondary structure of zein. The conformational change of zein was dependent on the lecithin concentration. The composition of α-helix, β-sheet and random coil was estimated by the K2D-SET4 software and the data were shown in the insert table in Fig 3. The secondary structural composition of native zein was 44% α-helix, 22% β-sheet and 34% random coil, respectively. This result was similar to that from Yong et al. [29], who reported that the α-helix content of α-zein was 46%. At zein to lecithin mass ratios of 20:1, 10:1 and 5:1, the α-helix content of zein was significantly (*p<0*.*05*) increased from 44% to 46%, 51%, and 58%, respectively. However, the β-sheet content was significantly (p<0.05) decreased from 22% to 20%, 17%, and 11%, respectively. This result might be attributed to the interaction between zein and lecithin. Mantovani, Fattori, Michelon, & Cunha [31] also observed that mixing soy lecithin to whey protein led to an increase in α-helix content from 20 to 23% and a decrease in β-strand content from 26 to 24%. This transition from native β-structure to non-native α-helical structure was a consequence of the protein-soy lecithin phospholipids interaction. Furthermore, continuing to increase the level of lecithin induced a decrease in α-helix content and an increase in β-sheet content, implying that the binding mode of zein and lecithin was changed. When the mass ratio of zein to lecithin was 1:2, the α-helix and β-sheet contents were 45% and 16%. Sun et al. [20] reported that the α-helix content decreased and β-sheet content increased with an increase of quercetagetin concentration, which revealed there existed the interaction between quercetagetin and zein.

### 3.4. FTIR

**Fig 4:**
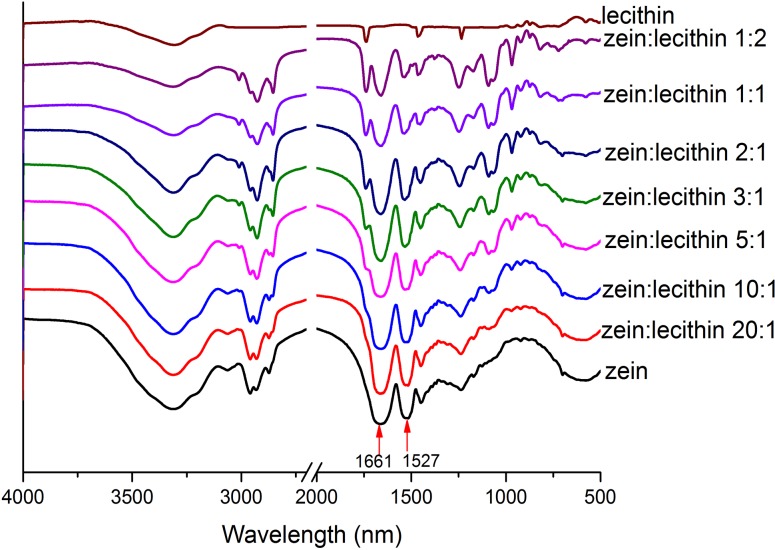
FTIR of zein and zein-lecithin composite colloidal nanoparticles.

The intermolecular interaction of zein and soy lecithin was characterized by FTIR. The representative spectra of zein, lecithin and their composite colloidal nanoparticles were shown in Fig 4. From Fig 4, it was found that the FTIR spectra of zein showed a prominent absorption peak at 3312 cm^-1^, which was resulted from the stretching vibration of N–H of amides A in amino acid residues [32]. Other two characteristic absorption peaks of zein at 1661 and 1517 cm^-1^ were amide I and amide II, respectively. The amide I band was associated with the C = O stretching and amide II absorption peak was due to bond C–N–H in-plane bending, the C–N and C-C stretching [33]. Both C = O and N-H bonds were involved in the hydrogen bonding between different elements contributing to the secondary structure of zein [7]. Compared with the spectrum of zein, the bands of amide I and amide II groups were shifted from 1661 and 1517 cm^−1^ to 1660 and 1531 cm^−1^, 1660 and 1533 cm^−1^, 1659 and 1534 cm^−1^, and 1659 and 1535 cm^−1^ in the spectra of zein and lecithin composite colloidal nanoparticles at the ratios of 10:1, 5:1, 3:1, and 2:1, respectively. These findings indicated there existed electrostatic interactions between zein and lecithin. Luo et al. [34] also reported that compared to native zein, the bands of amide I and amide II groups of α-tocopherol and zein composite nanoparticles were shifted from 1664 and 1550 cm^−1^ to 1660 and 1547 cm^−1^, respectively, suggesting that the electrostatic interactions occurred between α-tocopherol and zein. Moreover, zein and lecithin were both hydrophobic compounds, the hydrophobic interaction was another driving force involved during the formation of zein-lecithin complexes.

The FTIR spectrum of lecithin showed obvious peaks at 1740 (C = O stretching vibration), 1465 (C–H bending vibrations of the alkanes), and 1234 cm^−1^ (P = O stretching vibration) [35]. After the formation of zein-lecithin composite colloidal nanoparticles, the peak at 1234 cm^-1^ (P = O) was shifted to 1248 cm^-1^. This phenomenon might be due to some interactions between the peptide bond (CO-NH) of zein and the P = O bond of phospholipids during the formation of complexes [24].

### 3.5. DSC

**Fig 5:**
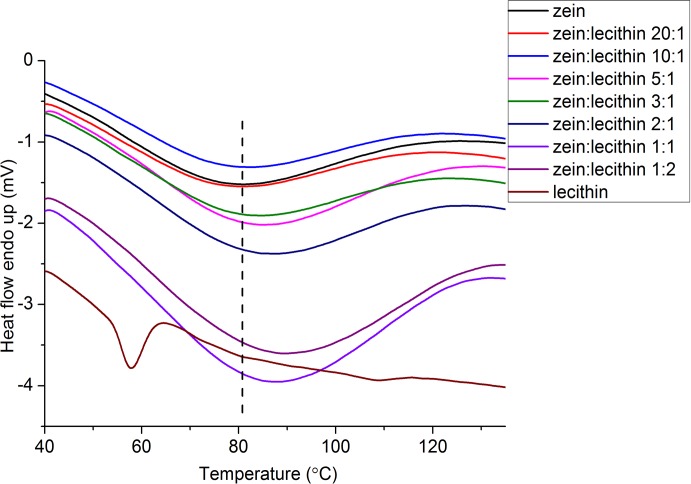
DSC thermograms of zein and zein-lecithin composite colloidal nanoparticles.

The DSC curves of zein, lecithin and zein-lecithin composite colloidal nanoparticles were presented in Fig 5. A negative heat flow represented an exothermic change. As shown in Fig 5, zein had a characteristic endothermic peak at 80.8°C. This result was consistent with the report of Luo et al. [34] who demonstrated that zein had an endothermic peaks at 73.2°C. The lecithin exhibited an endothermal peak at about 58.0°C, which was attributed to the fusion of its crystalline portion. The denaturation peak temperatures of zein and lecithin composite colloidal nanoparticles were 84.9, 85.2, 87.8, 88.1 and 89.4°C, at the zein and lecithin ratios of 5:1, 3:1, 2:1, 1:1 and 1:2, respectively, in the DSC thermograms. Compared with native zein, the denaturation peak temperature of zein and lecithin composite colloidal nanoparticles was increased with the rise of lecithin concentration. This result might be due to the hydrophobic attraction and electrostatic interaction between zein and lecithin, which made the structure of zein and lecithin composite colloidal nanoparticles more compact, requiring more energy to destroy the molecular structures. Zhang et al. [36] showed that the interaction between β-lactoglobulin and phospholipids, which was primarily due to the hydrophobic interaction, increased the thermostability of the protein.

### 3.6. AFM

**Fig 6:**
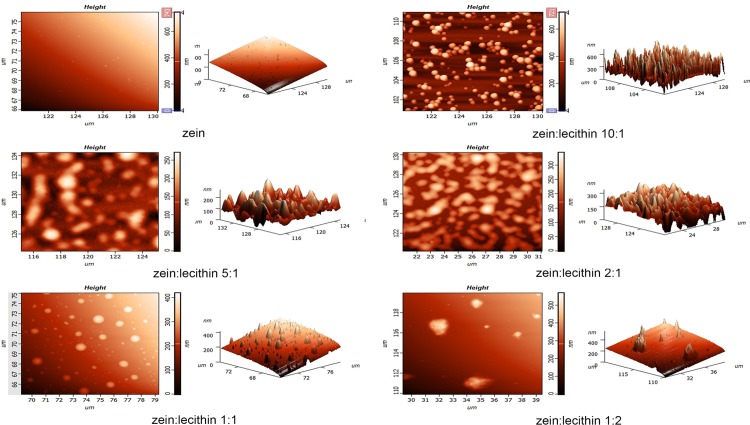
AFM images of zein and zein-lecithin composite colloidal nanoparticles.

The morphology of zein and zein-lecithin composite nanoparticles was studied using AFM. As shown in Fig 6, native zein nanoparticles exhibited typical nanospheres with smooth surface and homogeneous diameter. The average size of zein measured by AFM was 216.28 nm, which was apparently larger than that by Zetasizer. This result was similar to that reported by Sun et al. [3]. After adding lecithin, the morphology of zein-lecithin composite nanoparticles was obviously changed. When the mass ratio of zein to lecithin was 10:1, the morphology of zein-lecithin composite nanoparticles also had smooth surface, but was more uniform than that of zein nanoparticles. However, with the increase of lecithin concentration (zein to lecithin mass ratios of 5:1 and 2:1), the composite colloidal nanoparticles became irregular geometry shapes including partial long-bar shape, oval, dumbbell-like morphology and random geometry. And an average diameter of composite nanoparticle at the zein to lecithin ratio of 5:1 was elevated up to 260 nm. This result was consist with that of particle sizes featured by Zetasizer in Fig 1A. When the mass ratio of zein to lecithin reached to 1:1, the morphology of composite colloidal nanoparticles regained to smooth spherical shape. Interestingly, the zein-lecithin composite nanoparticles at the mass ratio of 1:2 exhibited a vesicle-like structure. Zhou et al. [24] also reported that insulin-lecithin complex fabricated by solvent evaporation method and also formed a reverse micelle-like structure.

### 3.7. Stability of Zein and Lecithin Colloidal Nanoparticles Dispersions

#### 3.7.1 Effect of salt

**Fig 7:**
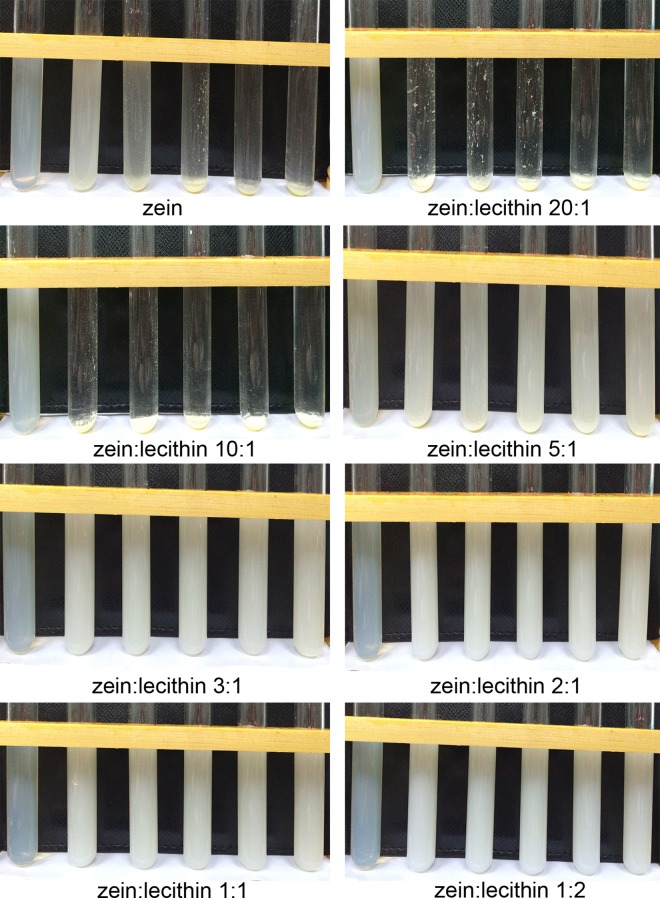
Effect of NaCl on zein and zein-lecithin composite colloidal nanoparticle dispersion stability.

It was important to determine the influence of ionic strength on the physical properties and stability of colloidal delivery systems, which experienced a variety of electrolytes in commercial products and human gastrointestinal tract. We therefore studied the influence of salt (NaCl) addition on the properties of zein and zein-lecithin dispersions in this study (Fig 7). The zein nanoparticles were highly sensitive to even low levels of salt, precipitated and formed a white sediment at the bottom of the test tubes when the NaCl concentration was as low as 25 mM. Continuing to increase the salt content, the sediment layer became thicker and the solution above it became clearer. This result was consistent with the previous researches. Patel et al. [2] and Hu [9] also reported zein nanoparticles were unstable in the presence of NaCl. When the salt was added, the electrostatic interactions between nanoparticles, which played an important role in stabilizing the zein nanoparticles against aggregation, were screened and the attractive interactions such as van der Waals force and hydrophobic effects were strong enough to overcome repulsive interactions, causing the extensive nanoparticle aggregation [2, 9, 23]. The samples of zein and lecithin complexes at the mass ratios of 20:1 and 10:1 were also unstable and formed sediment at the bottom of the test tubes and clearer supernatant after adding NaCl. As lecithin content increased, the zein and lecithin at the ratio of 5:1 showed little sediment at the bottom and creamy white solution above. When the zein and lecithin mass ratio was more than 5:1, the complexes of zein and lecithin at the mass ratios of 3:1, 2:1 1:1 and 1:2 were stable even at a higher level of salt (300 mM NaCl) and no precipitate occurred for these samples. At a higher lecithin content, the interaction between zein and lecithin composite colloidal nanoparticles was enough to maintain stability and prevent the aggregation. The instability of the zein nanoparticles in the presence of salt limited its application in food industry. The formation of zein and lecithin composite colloidal nanoparticles would be an appropriate choice to improve the salt stability.

#### 3.7.2 Effect of heat

**Fig 8:**
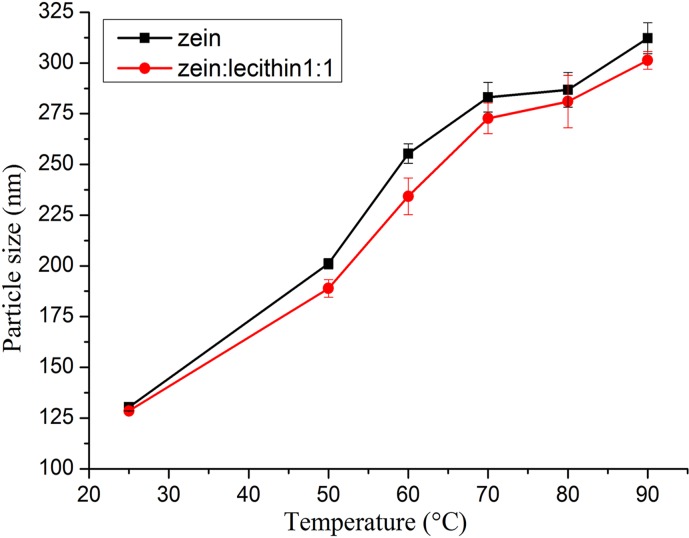
Effect of heating temperature on the mean particle size of zein and zein-lecithin composite colloidal nanoparticles (zein:lecithin = 1:1).

Colloidal delivery systems may experience heat treatment during the processing and manufacturing. Therefore, we also determined the influence of temperature on the stability of the zein and lecithin composite colloidal nanoparticles. The sizes of zein and zein-lecithin composite colloidal nanoparticles were determined after they were heated at different temperatures (50–90°C) (Fig 8). The sizes of zein and zein-lecithin composite colloidal nanoparticles were increased with the rise of temperature. The mean size of zein nanoparticles increased from 130 for the unheated sample to 312 nm for the sample heated at 90°C. Chen also reported that the size of zein nanoparticles increased at higher temperatures. Thermal treatment might lead to expose more reactive non-polar and sulfhydryl groups, which might increase hydrophobic attraction and disulphide bond formation within and between protein-coated particles and form larger particles [23]. There was also an appreciable increase (*p < 0*.*05*) in the size of zein-lecithin composite colloidal nanoparticles (zein to lecithin mass ratio of 1:1) with increased temperature, from 128 initially to 301 nm after heating at 90°C. These results indicated that the thermal treatment could destroy the interaction between zein and lecithin and result in reorganization of the molecules within the composite nanoparticles [2].

#### 3.7.3 Effect of pH

**Fig 9:**
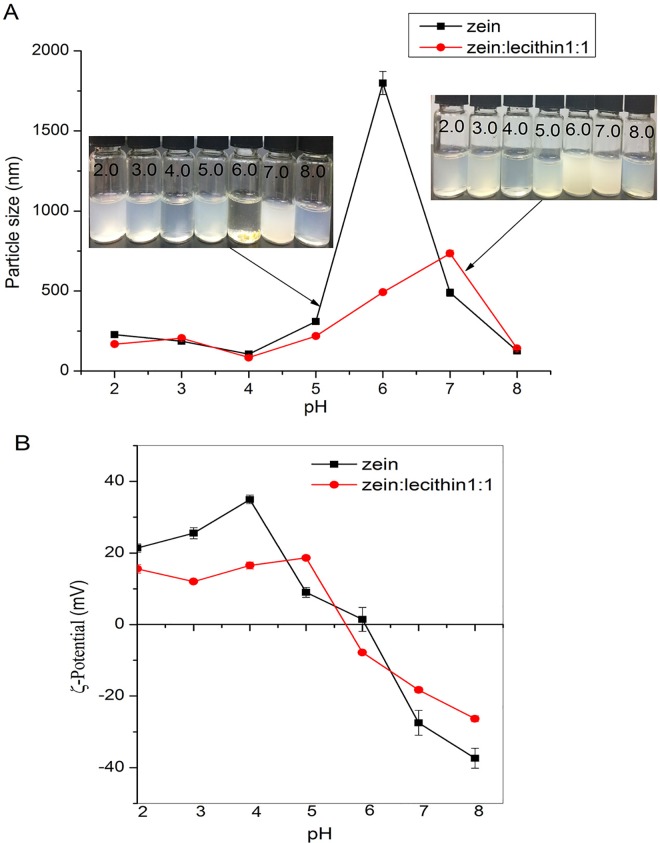
Effect of pH on the mean particle size (A) andζ-potential (B) of zein and zein-lecithin composite colloidal nanoparticles (zein:lecithin = 1:1).

The colloidal delivery systems used in commercial food and beverage products may undergo a range of pH conditions. In addition, they also experience different pH values as they go through the human gastrointestinal tract after oral ingestion. Therefore we investigated the influence of pH on the particle size andζ-potential of the zein and lecithin composite colloidal nanoparticles. In the absence of lecithin, the particle size of zein nanoparticles was relatively small at pH 2.0, 3.0 and 4.0 (Fig 9A). At pH 5.0, the colloidal dispersions showed a milky white appearance and the particle size and turbidity were slightly increased. At pH 6.0, a layer of sediment was observed at the bottom of the glass bottles with a slightly turbid layer above. This result can be ascribed to that the isoelectric point of zein is about pH 6.2 [10]. In the presence of lecithin, the composite colloidal nanoparticles were stable to aggregation from pH 2.0 to 5.0. At pH 6.0 and 7.0, there was an increase in particle size and the colloidal dispersions had a uniform milky white appearance. No precipitate occurred for these samples suggesting that zein and lecithin composite nanoparticles may be able to inhibit the particle aggregation, which normally occurs around pH 5.0 and 6.0.

Theζ-potential of zein and zein-lecithin composite nanoparticles at different pH were presented in Fig 9B. Theζ-potential of zein nanoparticles went from highly positive at low pH (2.0) to highly negative at high pH (8.0) with a point of zero charge around pH 6.1. It was consistent with the result of Shukla and Cheryan [37] who also reported that the isoelectric point of zein was around pH 6.2. The ζ-potential of zein and lecithin composite nanoparticles also presented highly positive at low pH (e.g., +21 mV at pH 2.0) and highly negative at high pH (e.g., -37 mV at pH 8.0). However, the point of zero charge of composite nanoparticles was around pH 5.8, which was lower than the isoelectric point of zein (pH 6.1). The reduction in the point of zero charge may be attributed to adsorption of lecithin on the surfaces of zein. Hu and McClements [10] also reported the non-ionic surfactant (Tween 80) reduced the ζ-potential of zein nanoparticles, which was attributed to binding of Tween 80 on surfaces of zein.

### 3.8. Potential Interaction Mechanism

From the results of dynamic light scattering, zeta-potential and turbidity measurement, fluorescence, CD analysis and DSC, as well as the morphological observation by AFM, it could be concluded that the interaction between zein and lecithin was dependent on the mass ratio of zein to lecithin and the change regulation suggested the existence of a critical mass ratio of zein to lecithin (5:1).

**Fig 10:**
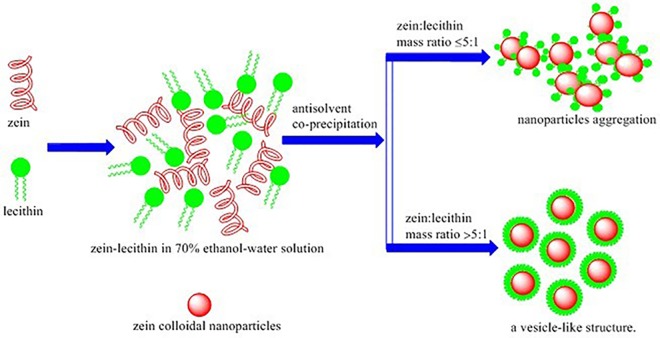
Illustration of the formation mechanism and possible structures of zein and lecithin composite colloidal nanoparticles.

The potential interaction mechanism between zein and lecithin was proposed in Fig 10. When lecithin concentration was below the critical mass ratio of zein to lecithin (5:1), the particle size and turbidity of zein and lecithin composite colloidal nanoparticles were significantly increased with the rise of lecithin level. The fluorescence emission intensity and CD ellipticity at 221 nm of the zein-lecithin complexes in 70% ethanol-water solution were also enhanced by the addition of lecithin. This result was related to the enhancement of the interaction between zein and lecithin such as hydrophobic effects, hydrogen bonding, as well as electrostatic interaction with the increasing of lecithin level, which led to the exposure of more tyrosyl groups on the molecular layer of zein, lower electrostatic repulsion and the micro- and nano-aggregation of zein-lecithin composite colloidal nanoparticles.

As lecithin concentration was increased beyond the critical mass ratio of zein to lecithin (5:1), zein-lecithin composite colloidal nanoparticles showed a smaller particle size, lower turbidity value and significant decreased fluorescence intensity. The excessive lecithin molecules were bound to the surface of zein-lecithin composite colloidal nanoparticles, forming vesicle-like structure and inhibiting the aggregation of composite nanoparticles.

## Conclusions

The presence of lecithin led to the significant change of the secondary structure and improved thermal and salt stability of zein. The changing magnitude was dependent on the mass ratios of zein to lecithin and suggested the existence of a critical mass ratio of zein to lecithin (5:1). At a lower lecithin concentration (zein to lecithin mass ratio ≤ 5:1), the zein and lecithin composite colloidal nanoparticles might aggregate due to the increased interaction at a higher proportion of lecithin. Nevertheless, continuing to increase the lecithin concentration might lead to excessive lecithin molecules covering on the surface of zein-lecithin composite colloidal nanoparticles, which would generate a vesicle-like structure, inhibit the aggregation and improve thermal and salt stability of composite colloidal nanoparticles. The zein-lecithin composite nanoparticles can expand the application of zein and lecithin, and are potentially used as foodgrade delivery systems to encapsulate and protect certain bioactive components.
